# Interactions Between Enteroviruses and the Inflammasome: New Insights Into Viral Pathogenesis

**DOI:** 10.3389/fmicb.2019.00321

**Published:** 2019-02-25

**Authors:** Xia Xiao, Jianli Qi, Xiaobo Lei, Jianwei Wang

**Affiliations:** ^1^NHC Key Laboratory of Systems Biology of Pathogens, Institute of Pathogen Biology, Chinese Academy of Medical Sciences and Peking Union Medical College, Beijing, China; ^2^Collaborative Innovation Center for Diagnosis and Treatment of Infectious Diseases, Hangzhou, China

**Keywords:** enteroviruses, NLRP3 inflammasome, 2B and 3D, 2A and 3C, pathogenesis

## Abstract

Enteroviruses (EVs) have emerged a substantial threat to public health. EVs infection range from mild to severe disease, including mild respiratory illness, diarrhea, poliomyelitis, hand, foot, and mouth disease, aseptic meningitis, and encephalitis. In the Asia-Pacific region, for example, one of the best studied enterovirus 71 (EV71) has been associated with pandemics of hand, foot, and mouth disease (HFMD) in children, particularly those under the age of five. Serious HFMD cases are associated with neurological complications, such as aseptic meningitis, acute flaccid paralysis, brainstem encephalitis, and have been associated with as many as 1000s of deaths in children and infants from 2008 to 2017, in China. More than 90% of laboratory confirmed deaths due to HMFD are associated with EV71. However, little is known about the pathogenesis of EVs. Studies have reported that EVs-infected patients with severe complications show elevated serum concentrations of IL-1β. The secretion of IL-1β is mediated by NLRP3 inflammasome during EV71 and CVB3 infection. Enteroviruses 2B and 3D proteins play an important role in activation of NLRP3 inflammasome, while 3C and 2A play important roles in antagonizing the activation of NLRP3 and the secretion of IL-1β. In this review, we summarize current knowledge regarding the molecular mechanisms that underlie the activation and regulation of the NLRP3 inflammasome, particularly how viral proteins regulate NLRP3 inflammasome activation. These insights into the relationship between the NLRP3 inflammasome and the pathogenesis of EVs infection may ultimately inform the development of novel antiviral drugs.

## Introduction

Enteroviruses are the members of *Picornaviridae*. International Virus Classification Committee classifies enteroviruses into 12 species according to their molecular structure and serotype analysis, including enterovirus A–J and Rhinovirus A–C. Five species (E–J) of enteroviruses infect only animals and the remaining seven species of enterovirus can cause various kinds of human diseases, such as infantile paralysis -caused by poliovirus (PV), myocarditis-caused by coxsackievirus B3 (CVB3), hand foot and mouth disease-caused by enterovirus 71 (EV71), coxsackievirus A16 (CVA16), CVA6 and other enteroviruses, respiratory disease-caused by enterovirus 68 (EV68), rhinovirus (Racaniello, 2013). In particular, the outbreak of hand foot and mouth disease every year, which seriously threatens the health of infants and young children, has brought enormous burden to our public health (Huang et al., 1999; Chan et al., 2003; AbuBakar et al., 2009; Yang et al., 2011; Xing et al., 2014). However, despite these risks, there are no effective antivirals for the treatment of enteroviruses (EVs) infection.

Enteroviruses are positive-sense, single-stranded RNA viruses. The genome of EVs is approximately 7,200∼7,400 nucleotides in length and contains a large open reading frame (ORF) which can be translated into a polyprotein of approximately 2,200 amino acids. Then, the polyprotein can be processed into four structural proteins and seven non-structural proteins by virus-encoded proteases. The structure proteins VP1, VP2, VP3, and VP4 are mainly involved in the formation of virus particles and the binding of virus to host cells. The non-structure proteins 2A, 2B, 2C, 3A, 3B, 3C, and 3D play pivotal roles in viral infection, viral replication, and innate immune response evasion (Racaniello, 2013). The non-structure proteins also mediate virus-host interactions, such as EVs 2A result in the shutoff the host RNA and protein synthesis by cleaving the eukaryotic translation initiation factor 4G (eIF4G) and the P220 subunit of the cap-binding protein complex, 2A and 3C can antagonize the innate immunity responses (Lei et al., 2016). It is therefore critical to understand the interactions between EVs and the host in order to elucidate and limit the pathogenesis of EVs.

Innate immunity is the first line of host defense against viral infection, characterized by the induction of type I interferon (IFN). Infection with EVs triggers host antiviral innate immune responses through pattern recognition receptors (PRRs), such as RIG-I-like receptors, Toll-like receptors, or NOD-like receptors (Feng et al., 2012; Ito et al., 2012; Kuo et al., 2013; Hsiao et al., 2014). For example, EV71 has evolved diverse strategies to evade host antiviral responses (Lei et al., 2010, 2013, 2014; Hung et al., 2011; Wang et al., 2013, 2015). During EV71 infection, EV71-encoded 2A protease directly cleaves MDA5 and RIG-1 to inhibit type I interferon (IFN) production (Feng et al., 2014). EV71 2A also targets the mitochondrial anti-viral signaling protein (MAVS, also named IPS-1, VISA, Cardif) and cleaves it at multiple sites (Wang et al., 2013). EV71 2A also directly inhibits the IFN signaling pathway by inducing the cleavage of type I IFN receptor (IFNAR1) to block the expression of interferon stimulated genes (ISGs) (Lu et al., 2012). EV71 3C protease inhibits the RIG-I and MAVS interaction and induces the cleavage of TIR-domain-containing adaptor-inducing IFN-β (TRIF), interferon regulatory factor 7/9 (IRF7/9), and the transforming growth factor-β-activated kinase (TAK1) complex (Hung et al., 2011; Lei et al., 2011, 2013, 2014). Together, the EV71 2A and 3C proteases play critical roles in inhibiting the production of IFN and the IFN downstream signaling pathway, thereby antagonizing the host antiviral response.

The inflammasome pathway is also an integral component of the innate immune response against microbes (Martinon et al., 2002). In this review, we discuss the NLRP3 activation pathway that is induced by enterovirus infection, the role of NLRP3 activation in the pathogenesis of EVs infection, and the role of EVs non-structure proteins in the activation and/or inhibition of the NLRP3 inflammasome.

## Signaling Pathways That Promote Inflammasome Activation

The inflammasome complex consists of several proteins, including apoptosis-associated speck-like protein containing CARD (ASC) protein, caspase proteases, and a family of NOD-like receptor (NLR) proteins (such as NLRP3) or IFI200 family proteins (such as AIM2) (Martinon et al., 2002; Muruve et al., 2008; Lugrin and Martinon, 2018). To date, five types of inflammasomes have been identified: the NLRP1 inflammasome, the NLRP3 inflammasome, the NLRC4 inflammasome, the AIM2 inflammasome, and the Pyrin inflammasome (Man and Kanneganti, 2015). The NLRP3 inflammasome has been extensively studied because of its possible involvement in several human diseases and microbial infections. For example, the NLRP3 inflammasome plays a role in promoting hyperinflammation and disease during severe IAV infection (Allen et al., 2009; Ichinohe et al., 2009; McAuley et al., 2013; Kuriakose and Kanneganti, 2017). The NLRP3 inflammasome is also involved in EV71 and CVB3 infection (Wang et al., 2014, 2015, 2017, 2018a; Li et al., 2017; Miteva et al., 2018), suggesting a potential role for the NLRP3 inflammasome in viral pathogenesis.

### The NLRP3 Inflammasome

NLRP3 is a member of the NLR protein family. NLR family proteins contain an N-terminal pyrin domain (PYD), a central nucleotide-binding and oligomerization domain (NACHT) (also known as a NOD domain), and C-terminal leucine-rich repeats (LRRs) (Kufer et al., 2005). The N-terminal domain of NLRP3 mediates signal transduction via direct interactions with other CARD- or PYD-containing proteins. The central NACHT domain of NLRP3 mediates oligomerization and serves as a scaffold protein for inflammasome assembly, which is believed as an important step to activate inflammasome. The C-terminal LRRs of NLRP3 are thought to serve as a ligand sensor (Chen and Ichinohe, 2015). Upon activation, NLRP3 oligomerizes via homotypic interactions between NACHT domains and presents clustered PYD domains to recruit ASC. The ASC adaptor protein then binds to NLRP3 protein through a PYD-PYD interaction. The resulting clusters of ASC protein directly recruit pro-caspase-1 via a CARD-CARD interaction to form a large cytosolic NLRP3 inflammasome complex. Pro-caspase 1 then induces proximity-induced auto-cleavage of caspase-1 (Lu et al., 2014). The activated caspase-1 subsequently processes pro-IL-1β and pro-IL-18 into their active forms, which are secreted to the extracellular space and initiate downstream inflammation.

### Two Signals for Activating the NLRP3 Inflammasome Pathways

Activation of the NLRP3 inflammasome requires at least two signals. Signal I, known as the priming signal, activates the NF-κB signaling pathway through the activation of tumor necrosis factor (TNF) or PRRs, such as the TLRs and NLRs, and further induces the expression of pro-IL-1β, pro-IL-18 and NLRP3 (Bauernfeind et al., 2009; Franchi et al., 2009; He et al., 2016). During RNA virus infection, signal I is evoked by the recognition of viral RNA by toll-like receptors (TLRs) (Pothlichet et al., 2013) or retinoic acid-inducible gene-I (RIG-I)-like receptors (Poeck et al., 2010). The priming of the NLRP3 inflammasome is regulated by several factors. The induction of NLRP3 expression during priming is regulated by FAS-associated death domain protein (FADD) and caspase-8 (Allam et al., 2014; Gurung et al., 2014). Anaplastic lymphoma kinase (ALK), which regulates the activation of NLRP3 inflammasome in macrophages and mediates NF-κB activation, is required for the priming step of NLRP3 upregulation (Zhang et al., 2018). Pellino2, an E3 ubiquitin ligase, has also been demonstrated to promote the ubiquitination of NLRP3 during the priming phage of activation, and IRAK1 plays a negative role in activation of NLRP3 inflammasome through interacting with Pellino2 (Humphries et al., 2018). Emerging research has shown that priming the NLRP3 inflammasome through signal I involves transcription-dependent and post-translation-dependent manners. The adaptor MyD88 and the downstream kinase IRAK1 (IL-1 receptor-associated kinase 1) and IRAK4 have been identified to active NLRP3 through transcriptional manner, whereas the adaptor TRIF and IRAK1 play roles in the post-transcriptional manner (Bauernfeind et al., 2009; Fernandes-Alnemri et al., 2013; Lin et al., 2014).

Signal II, also known as the activating signal, is mediated by numerous pathogen-associated molecular patterns (PAMPs) or by danger-associated molecular patterns (DAMPs). Signal II is responsive to a wide range of stimuli, such as adenosine triphosphate (ATP), monosodium urate (MSU), pore-forming toxins, viral RNA or DNA, and ultraviolet radiation (Pelegrin and Surprenant, 2007; He et al., 2016; Yu et al., 2017). Signal II promotes the assembly of ASC and pro-caspase-1, leading to the activation of the NLRP3 inflammasome complex (Afonina et al., 2017). Several molecular and cellular events have been identified as stimuli for NLRP3 inflammasome activation, including K^+^ efflux, Ca^2+^ signaling, reactive oxygen species (ROS), nigericin, mitochondrial dysfunction, lysosomal rupture, viroporins, and some viral functional proteins (Perregaux and Gabel, 1994; Lee et al., 2012; Katsnelson et al., 2015). Potassium (K^+^) efflux is thought to be an essential mechanism for ATP-induced NLRP3 inflammasome activity. A reduction in cytosolic K^+^ is necessary for the induction of NLRP3 inflammasome assembly (Perregaux and Gabel, 1994). Consistent with this, TWIK2, a K^+^ efflux channel in macrophages, plays a fundamental role in activating the NLRP3 inflammasome and consequently mediates inflammation (Di et al., 2018). Calcium-sensing receptor (CASR) can also activate the NLRP3 inflammasome by increasing intracellular Ca^2+^ and decreasing cellular cyclic AMP (cAMP). Increased cytoplasmic Ca^2+^ promotes the assembly of inflammasome components and the release of IL-1β (Lee et al., 2012). Importantly, the role of decreased K^+^ efflux in activating NLRP3 inflammasome signaling is not independent of changes in cytosolic Ca^2+^ (Katsnelson et al., 2015).

Mitochondrial dysfunction can also cause NLRP3 inflammasome activation. Treatment with the mitochondria complex I inhibitor rotenone, which leads to a loss of mitochondrial membrane potential, increases the NLRP3-dependent IL-1β secretion (Zhou et al., 2011). During injury, mitochondrial DNA (mtDNA), ATP, and mitochondrial reactive oxygen species (mtROS) have also been shown to promote NLRP3 inflammasome activation, either directly or via specific receptor such as FPR1 and P2RX7 (Cano Sanchez et al., 2018). Recently, it was found that newly synthesized mitochondrial DNA is crucial for NLRP3 inflammasome activation and that this process is dependent on the mitochondrial deoxyribonucleotide kinase CMPK2 (Zhong et al., 2018). Viroporins such as human rhinovirus (HRV) 2B protein (Triantafilou et al., 2013b), respiratory syncytial virus (RSV) SH protein (Triantafilou et al., 2013a), influenza A virus (IAV) M2 protein (Ichinohe et al., 2010), encephalomyocarditis virus (EMCV) 2B protein (Ito et al., 2012), severe acute respiratory syndrome coronavirus (SARS-CoV) envelope (E) protein (Nieto-Torres et al., 2015), SARS-CoV accessory protein open reading frames (SARS 3a) oligomerizes (Yue et al., 2018), and the p7 viroporin of hepatitis C virus (HCV) (Farag et al., 2017) have also been reported to active the NLRP3 inflammasome by disturbing intracellular ionic concentrations, particularly through potassium efflux, calcium efflux, and by changing cellular pH.

Several studies have also reported that plasma vRNA may bind to NLRP3 and subsequently activate it. The DExD/H-box helicase (DHX) family members have been shown to participate in the process by which vRNA activates the NLRP3 inflammasome (Poeck et al., 2010; Mitoma et al., 2013; Pothlichet et al., 2013; Li et al., 2015). DHX33, DDX19A, and DDX58 have been reported to regulate NLRP3 inflammasome activation through combining directly with vRNA to bind to NLRP3 (Poeck et al., 2010; Mitoma et al., 2013; Chakrabarti et al., 2015).

### GSDMD-Mediated Pyroptosis Is Critical for IL-1β Secretion

In addition to inducing the activation and secretion of IL-1β and IL-18, activated caspase-1 triggers pyroptosis, an inflammatory form of programmed cell death (Jorgensen and Miao, 2015). Pyroptosis is characterized by cell swelling, membrane blebbing, and the disintegration of the cell membrane (Shi et al., 2017). In general, pyroptosis is believed to protect multicellular host organisms against invasive pathogenic bacteria and microbial infections (Mathur et al., 2018). During infection, pyroptosis damages the intracellular replication niches of pathogens, promotes the release the pro-inflammatory cytokines, such as IL-1β and TNFα, and triggers inflammation (Miao et al., 2010, 2011; Shi et al., 2017). But how is the pyroptosis program activated? Shi et al. (2015) recently demonstrated that gasdermin D (GSDMD) is a key pyroptosis substrate of inflammatory caspases. The gasdermin family plays a central role in inflammasome signaling and cell pyroptosis. The deficient of GSDMD in ASC-reconstituted RAW264.7 cells impaired IL-1β and IL-18 secretion upon LPS stimulation (He et al., 2015). With the LPS stimulation, the level of IL-1β secreted by primary *Gsdmd*^-/-^ bone marrow-derived macrophages (BMDMs) is lower than that secreted by wild type BMDMs (Shi et al., 2015). Further study showed that in *Gsdmd*^-/-^ BMDMs, caspase-1 autoprocessing occurs normally upon activation, while the extracellular secretion of both pro-caspase-1 and the mature caspase-1 is severely inhibited. Meanwhile, in the cytosol of *Gsdmd*^-/-^ BMDMs, intact caspase-1 autoprocessing and IL-1β maturation were also observed upon canonical inflammasome activation. These data illustrate that GSDMD plays an important role in the release of mature IL-1β without affecting IL-1β maturation (He et al., 2015; Shi et al., 2015). GSDMD also can be cleaved by caspase-11 in non-canonical inflammasome signaling (Kayagaki et al., 2015). The N-terminus of GSDMD (the product of GMDMD cleaved by caspase-1) is sufficient to induce pyroptosis by directly relocating to the membrane and inducing pore formation (He et al., 2015; Shi et al., 2015; Lei et al., 2017).

## Activation of the NRLP3 Inflammasome by Enterovirus Infection

The NLRP3 inflammasome has been extensively investigated in RNA viral infection (Kanneganti et al., 2006; Allen et al., 2009; Kuriakose and Kanneganti, 2017). A variety of viral infections can induce the activation of the NLRP3 inflammasome, including IAV, hepatitis C virus, coxsackievirus B3 (CVB3), EV 71, HRV and EMCV (Allen et al., 2009; Ito et al., 2012; McAuley et al., 2013; Negash et al., 2013; Triantafilou et al., 2013a; Wang et al., 2014, 2015). During infection, viroporins, which are a group of virus-encoded proteins that enhance the permeability of host cell membrane, and some viral functional proteins play an important role in stimulating the NLRP3 inflammasome through Signal II. These proteins include IAV M2, IAV PB1-F2, EV 2B, RSV SH, HCV p7, and EV71 3D (Ichinohe et al., 2010; Ito et al., 2012; McAuley et al., 2013; Triantafilou et al., 2013a,b; Farag et al., 2017; Wang et al., 2017). Almost all enteroviruses can activate the NLRP3 signaling pathway (Table 1).

**Table 1:** Modulation of the NLRP3 inflammasome in by enteroviral factors.

Virus	Viral/host factors	Regulating mechanism	Reference
EV71	2A	Cleaves NLRP3 to inhibit the activation of the NLRP3 inflammasome and reduces IL-1β release	Wang et al., 2015
	3C	Cleaves NLRP3 to inhibit the activation of the NLRP3 inflammasome and reduces IL-1β release	Wang et al., 2015
	3D	Forms a “3D-NLRP3-ASC” complex and then binds to pro-caspase-1, forming caspase-1, which mediate the secretion of IL-1β	Wang et al., 2017
CVB3	ROS, K^+^ efflux	Activate the NLRP3 inflammasome	Wang et al., 2014
	Cathepsin B	Activates the NLRP3 inflammasome	Wang et al., 2018b
EV71 and CVB3	Pyroptosis	Activates caspase-1	Wang et al., 2018c
EV71	3C	Cleaves GSDMD	Lei et al., 2017
CVB3	3C	Cleaves NLRP3 Cleaves RIP1 to inactive the NLRP3 inflammasome	Wang et al., 2018a
EMCV, PV, and EV71	2B	Induces NLRP3 redistribution	Ito et al., 2012
HRV	2B	Triggers the activation of NLRP3 and NLRC5 inflammation and the secretion of IL-1β	Triantafilou et al., 2013b


### Enterovirus Infection Induces NLRP3 Activation

The activation of the NLRP3 inflammasome is an important part of innate immunity against pathogen infection. Triantafilou et al. (2013b) show that HRV infection induces IL-1β secretion and caspase-1 activation in bronchial cells in NLRP3 and NLRC5 dependent manner. Recent studies have shown that EV71 infection induces the production and secretion of IL-1β and IL-18 in human monocytic THP1 cells, mouse bone marrow-derived cells (BMDCs), human macrophages, and peripheral blood mononuclear cells (PBMCs) (Wang et al., 2015, 2017). Furthermore, the EV71-induced secretion of IL-1β is significantly decreased in NLRP3-, ASC-, and caspase-1-deficient cells, indicating that EV71 infection activates the NLRP3 inflammasome and induce the maturation and secretion of IL-1β (Wang et al., 2015). EV71 entry and replication are required for the activation of the NLRP3 inflammasome because chlorpromazine, an entry inhibitor, and rupintrivir, a replication inhibitor, have been shown to inhibit EV71-induced IL-1β maturation and secretion (Wang et al., 2015). Wang et al. (2017) also found that EV71 replication and protein synthesis, but not EV71 genomic RNA, are required for the activation of IL-1β in macrophages and PBMCs, because ultraviolet- or heat-inactivated EV71 did not induce the secretion of IL-1β. In addition, treatment with EV71 viral genomic RNA is able to stimulate the expression of pro-IL-β by activating NF-κB but not affecting the secretion of IL-1β (Wang et al., 2017).

During early infection with EV71 in mice, brain tissue from NLRP3-inflammasome-deficient showed decreased levels of IL-1β. This early response against EV71 infection seems to be beneficial to the host because NLRP3-, ASC-, and caspase-1-deficient mice show more severe disease than WT mice (Wang et al., 2015). Due to the higher EV71 viral load in these deficient mice at late time, the levels of inflammasome-independent pro-inflammatory cytokines and chemokines in these deficient mice increased more quickly than in wild type mice. EV71-caused symptoms in these-deficient mice were worsen than in WT mice, indicating that NLRP3 inflammasome plays a protective role against EV71 infection (Wang et al., 2015). At the early stage of CVB3 infection, the NLRP3 inflammasome was also activated, as evidenced by increased gene expression of NLRP3 and pro-IL-1β and by the secretion of IL-1β and activated caspase-1 (Wang et al., 2014). When inflammasome activation is inhibited or deficient, the symptoms caused by CVB3 infection are also reduced (Wang et al., 2014). NLRP3 inflammasome activation in response to CVB3 infection is dependent upon ROS production and K^+^ efflux (Wang et al., 2014). In addition, Cathepsin B (CatB) has been shown to play an important role in CVB3-induced inflammasome activation and pyroptosis (Wang et al., 2018b).

### Activation of the NLRP3 Inflammasome by Enteroviral 2B Protein

It has been reported that the inflammasome pathway and its downstream cytokines play key roles in enterovirus-induced inflammation, but the mechanism by which this enterovirus-induced inflammation is triggered remains unclear. Triantafilou et al. (2013b) demonstrated that the HRV-2B protein enhanced the permeability of the host cell membrane and mediated the activation of the inflammasome. Enterovirus 2B protein has been reported to be a virally encoded viroporin that changes intracellular Ca^2+^ homeostasis and stimulates NLRP3- and NLRC5-dependent inflammasome activation by directly co-localization with NLRP3 and NLRC5 in the Golgi (Triantafilou et al., 2013b). Transfecting bronchial cells with 2B protein causes a decrease in the calcium-filling state of the ER and Golgi and induces an increase in cytosolic Ca^2+^, which is necessary for activation of inflammasome. Other picornaviral 2B proteins are also able to increase the permeability of the membrane structure and to regulate the Ca^2+^ balance, both of which are important in regulating inflammasome activation. Ito et al. (2012) showed that EMCV 2B protein is sufficient to activate the NLRP3 inflammasome by stimulating Ca^2+^ flux from the ER to the cytosol. Poliovirus and EV71 2B proteins also induce the redistribution of NLRP3 (Ito et al., 2012). However, Wang et al. found that EV71 2B protein did not induce the secretion of IL-1β *in vitro* in a 293T cell system into which plasmids encoding NLRP3, ASC, pro-caspase-1, pro-IL-1β and EV71 2B respectively were transfected (Wang et al., 2017). The coxsackievirus 2B protein can also form membrane-integral pores and can lead to an increase in the efflux of Ca^2+^ from the ER stores (de Jong et al., 2006). However, whether EV71 2B activates the NLRP3 inflammasome remains to be determined. These questions call for further study.

### Activation of the NLRP3 Inflammasome by EV71 3D Protein

**FIGURE 1:**
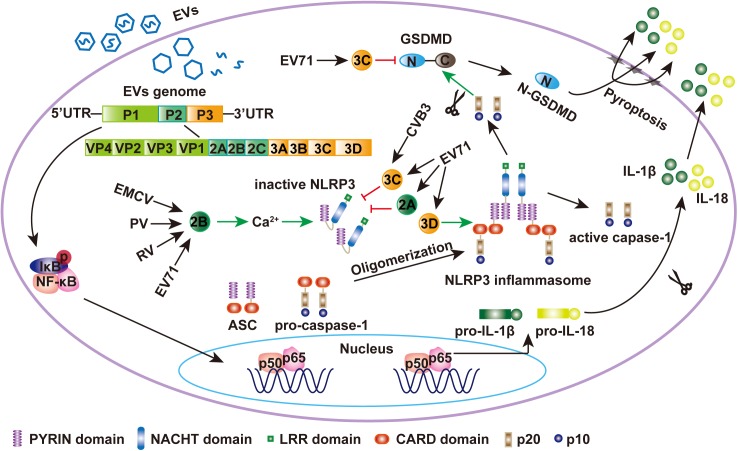
EV71 regulates the NLRP3 inflammasome. During EV71 infection, viral RNA is detected by PRRs and NF-κB is activated, which leaded to the upregulation of NLRP3, pro-IL-1β, and pro-IL-18. Meanwhile, EV71 3D protein binds to the LRR domain of NLRP3, which subsequently interacts with the PYRIN domain of ASC to form a “3D-NLRP3-ASC” ring-like inflammasome complex (Wang et al., 2017). The CARD domain of pro-caspase-1 then interacts with the CARD domain of ASC to form activated caspase-1, which mediates the maturation and secretion of IL-1β and IL-18. EMCV 2B was sufficient to activate NLRP3 inflammasome. 2B of poliovirus and EV71 also induces the NLRP3 redistribution (Ito et al., 2012). The EV71 and CVB3 3C proteases and EV71 2A can inhibit NLRP3 inflammasome activation by cleaving NLRP3 (Wang et al., 2015, 2018a). Activated caspase-1 also cleaves GSDMD at D275-G276 to produce N_1-275_-GSDMD, which is sufficient to induce pyroptosis by embedding itself directly within the membrane and inducing pore formation to release IL-1β. EV71 3C can cleave GSDMD at the Q193-G194 pair to produce N_1-193_-GSDMD, which does not induce cell pyroptosis (Lei et al., 2017).

By reconstructing the NLRP3 inflammasome *in vitro*, Wang and colleagues found that EV71 3D, an RNA-dependent RNA polymerase (RdRp), stimulates the activation of the NLRP3 inflammasome and induces the secretion of IL-1β. When 3D was co-expressed with the components of the NLRP3 inflammasome in 293T cells, the secretion of IL-1β in cell supernatants was increased. EV71 3D also induces the formation of the ASC pyroptosome and promotes the cleavage of pro-caspase-1. Importantly, this activity is not dependent on the RdRp activity of 3D. Meanwhile, 3D activates the NLRP3 inflammasome by directly interacting with the NACHT and LRR domains of NLRP3, forming a 3D-NLRP3-ASC ring-like structure to facilitate the assembly of the inflammasome. These data support the model that EV71-3D plays a critical role in the activation of the inflammatory response (Figure 1) (Wang et al., 2017). There are no evidences reveal that other enteroviral 3D proteins participate in the activation of inflammasome.

## The Role of the NLRP3 Inflammasome in Enteroviral Pathogenesis

As described above, EV71 infection induces the activation of NLRP3 inflammasome in human and mouse monocyte cells. In a mouse model, the NLRP3 inflammasome plays a protective role against EV71 infection. Mice that are defective in inflammasome components, including NLRP3, ASC, or caspase-1, had more severe symptoms than that of WT mice (Wang et al., 2015). Therefore, NLRP3 inflammasome activation is critical in defending the host from enterovirus infection.

The activation of the NRLP3 inflammasome releases IL-18, another important cytokine. IL-18 belongs to the IL-1 family and is synthesized as precursor protein that is mainly produced by monocytes/macrophages in response to PAMP/DAMP recognition by PRRs (Pothlichet et al., 2013). Li et al. (2017) found that IL-18 plays a protective role against EV71 infection *in vivo*. Like IL-1β, the IL-18 precursor has no biological activity and needs to be cleaved to form the mature cytokine. Our previous study reported that IL-18 is secreted from monocytic THP-1 cells in response to EV71 infection (Wang et al., 2015), indicating that IL-18 may be involved in the pathogenesis of EV71. Consistent with this observation, Wang et al. (2018c) showed that the expression level of IL-18 in EV71-infected HeLa cells was also elevated relative to mock-infected cells. In SH-SY5Y bone marrow neuroblastoma cells, IL-18 mRNA and protein levels were also increased after EV71-infection (Zhu et al., 2018). In these cell lines, the cleavage and maturation of IL-18 are dependent on the activation of caspase-1. Furthermore, studies suggest that serum levels of IL-18 are significantly increased in EV71-infected patients, especially in severe cases (Lin et al., 2003; Zhang et al., 2013). These findings indicate that cytokine responses may play a key in the pathogenesis of EV71, a model that is corroborated by *in vivo* data from mice. Li et al. (2017) showed that *IL-18*^-^*^/^*^-^ mice were more sensitive to EV71 infection than WT mice, suggesting that IL-18 plays a protective role against EV71 infection in mice. Recombinant IL-18 significantly decreases the mortality of mice infected with EV71 compared to control mice (Li et al., 2017).

## EV71 3C and 2A Inhibit NLRP3 Inflammasome Activation

### EV71 3C and 2A Cleave the NLRP3

The 3C and 2A proteases are important non-structural enterovirus proteins with protease activity. Previous studies have demonstrated that 3C and 2A cleave many host factors related to translation, mRNA processing and polyadenylation, cell apoptosis, the innate immune response, and more (Krausslich et al., 1987; Joachims et al., 1999; Kuo et al., 2002; Weng et al., 2009; Lei et al., 2010; Hung et al., 2011). Importantly, EV71 3C and 2A cleave several components of the innate immune response, such as TRIF, IRF9, IRF7, the TBK1 complex, and MAVS to evade innate immunity (Hung et al., 2011; Lei et al., 2011, 2013, 2014; Wang et al., 2013). Recently, work in our laboratory showed that the 3C and 2A proteases cleave NLRP3 protein at residues Q225-G226 and G493-L494, respectively. This cleavage inhibits the activation of the NLRP3 inflammasome in 293T cells bearing an NLRP3 inflammasome that was reconstituted by transfection with plasmids expressing NLRP3, ASC, pro-caspase-1, and pro-IL-1β (Wang et al., 2015). Similarly, Wang et al. (2017) reported that co-expression of the EV71 2A or 3C proteases with NLRP3 inflammasome components decreases the production and secretion of IL-1β *in vitro*. They also showed that the cleavage of caspase-1 is inhibited by 2A and reduced by 3C and that the expression of NLRP3 is reduced by both 3C and 2A. The 2A or 3C-induced degradation of NLRP3 may be important for IL-1β and IL-18 release from EV71 infection. Recent studies have shown that CVB3 infection results in the degradation of NLRP3 and its upstream RIP1/RIP3 protein by its proteinase 3C and leads to the inactivation of the NLRP3 inflammasome (Wang et al., 2018a). Thus, enteroviruses may directly antagonize the function of NLRP3 inflammasome by inducing the cleavage of NLRP3.

### 3C Induces the Cleavage of GSDMD

Gasdermin D is activated by inflammasome-associated inflammatory caspases, including human caspase-1, human caspase-4, human caspase-5, and mouse caspase-11. Activated caspase-1 and caspase-11 cleave GSDMD at the D275-G276 pair in human and mouse GSDMD (Shi et al., 2015) and produce N-terminal and C-terminal fragments of GSDMD. The N-terminal fragment of GSDMD is required to induce cell pyroptosis and IL-1β secretion. An over-expressed EV71 3C protease associates with GSDMD and induces the cleavage of GSDMD at the Q193-G194 pair (Lei et al., 2017). This cleavage is also observed in EV71-infected 293T cells in which GSDMD is over-expressed. The N-terminal 1–193 aa fragment of GSDMD (generated by 3C) is shorter than that induced by caspase-1, and it is unable to induce pyroptosis. Amino acids T239 and F240 in the 193–275 region of GSDMD are the key sites for pyroptosis induced by the N-terminal 1–275 fragment of GSDMD. In contrast to the 1–275 GSDMD fragment, the 1–193 fragment GSDMD generated by 3C cannot inhibit EV71 production. However, it is not clear which of the two cleavage fragment plays a leading role during EV71 infection. Ultimately, understanding the exact function of these two proteases and their cleavage products will be important for determining the role of GSDMD in the pathogenesis of EV71.

## Conclusion and Perspectives

Up to date studies indicate that enteroviral infection activate the NLRP3 inflammasome, which plays a key role in the innate immune response to control viral infections. NLRP3-inflammasome-deficient mice exhibit higher morbidity rates than their wild type counterparts. IL-18 also protects mice against EV71 infection. Cell death is also an important component of host defense against viral infection. Recently, pyroptosis was induced in EV71-infected epithelial and neuronal cells, indicating that pyroptosis is involved in the pathogenesis of EV71. To overcome these host responses, enteroviruses have evolved strategies to inhibit the activation of the NLRP3 inflammasome and to the limit induction of pyroptosis by cleaving the NLRP3 or GSDMD. Because severe cases of HFMD are associated with extreme inflammatory responses, an effective NLRP3 inhibitor may be able to improve patient outcomes by limiting enterovirus-induced inflammation. The role of pyroptosis in the pathogenesis of enteroviruses is not yet fully clear, whether GSDMD or other Gasdermins-mediated pyroptosis is critical in the pathogenesis of EV71 requires further study. Ultimately, detailed insights into inflammatory responses and pyroptosis in the pathogenesis of enteroviruses can inform new diagnosis and treatment options for EVs infection.

## Author Contributions

XL and JW provided the concept and study information, revised the manuscript, and provided the final version. XX, JQ, XL, and JW drafted the manuscript. All authors approved the final version of the manuscript.

## Conflict of Interest Statement

The authors declare that the research was conducted in the absence of any commercial or financial relationships that could be construed as a potential conflict of interest.
